# Liaison of Sugar Control With Time to Sputum Culture Conversion in Multi-Drug Resistant Tuberculosis

**DOI:** 10.7759/cureus.9395

**Published:** 2020-07-26

**Authors:** Saira Jafri, Naseem Ahmed, Nausheen Saifullah, Nadia Jawad, Intisar A Siddiqui

**Affiliations:** 1 Pulmonology, Jinnah Postgraduate Medical Centre, Karachi, PAK; 2 Chest Medicine, Jinnah Postgraduate Medical Centre, Karachi, PAK; 3 Pulmonology, Jinnah Post Graduate Medical Centre, Karachi, PAK; 4 Medicine, Jinnah Postgraduate Medical Centre, Karachi, PAK; 5 Research & Bio-Statistics, Imam Abdulrahman Bin Faisal University, Dammam, SAU

**Keywords:** hba1c, glycemic control, sputum culture conversion, multi-drug resistant tb

## Abstract

Background

Many elements have been studied repeatedly that influence time to sputum culture conversion in multi-drug resistant tuberculosis (MDR-TB).^ ^Deranged sugars not only hamper one’s infection contesting ability but also increase the chances of drug resistance. Our aim was to establish whether or not glycemic control alters MDR-TB treatment outcome.

Methods

A prospective cohort study was conducted at the TB Clinic of Jinnah Postgraduate Medical Center, Karachi, Pakistan. Newly diagnosed MDR-TB cases were started on WHO-recommended treatment regime. HbA1c (hemoglobin A1c or glycated hemoglobin) was tested at the start of treatment irrespective of the previous diabetic status. Sputum samples, 30 days apart, were taken during the initial phase of the MDR TB treatment until two consecutive samples showed conversion. Pearson's correlation coefficient was calculated to see the link between time to sputum culture conversion and HbA1c.

Results

Among 47 patients, 19 (40.4%) new cases, whereas 28 (59.8%) were previously treated for drug-sensitive TB. Our 39 patients converted during six months, of which 18 (46%) converted in one month, 14 (35.9%) in two months, 6 (15.4%) in three months, and only 1 in five months. Mean time to sputum culture conversion was 1.77 ± 0.9 months. There was a slightly negative correlation between HbA1c and sputum culture conversion time (r = -0.075, p = 0.649).

Conclusions

Sugar control does not affect sputum culture conversion in MDR-TB when an optimal treatment regime is applied.

## Introduction

Tuberculosis (TB) remains one of the leading infectious causes of morbidity and mortality worldwide [1]. Drug-resistant TB (DR-TB) is a major challenge to national TB control programs (NTPs) [2]. In 2018, there were an estimated 484,000 incident DR-TB patients worldwide. Pakistan is among the world's high-burden countries for drug-susceptible (DS) and DR-TB, with an estimated 28,000 new DR-TB patients (4.2% and 16% of new and re-treatment patients, respectively) in 2018. The treatment success rate for DR-TB in Pakistan was 64% in 2018, which is higher than the global treatment success rate (56%) [3].

Diabetes mellitus (DM), a non-communicable disease, is on the rise. The 2016 Global Diabetes Report stated that 422 million people were affected by DM [4]. A recent study estimated that by 2030 Pakistan will have the fifth largest number of type 2 DM patients [5].

Linkage between DM and DS-TB treatment outcome has been investigated time after time. It was seen that DM patients with active TB have a greater bacillary load at presentation, which results in longer time to culture conversion and prolonged treatment [6]. Chiang et al. stated that poor glycemic control is related to poor TB treatment outcomes [7].

In contrast, Salindri et al. in their study found that 90.9% diabetic and 82.9% non-diabetic patients achieved sputum culture conversion in MDR-TB [8]. This dearth of facts led us to the rationale of our study, which was to see the effect of spot glycemic control (unrelated to the presence or absence of DM) on time to sputum culture conversion in patients presenting with MDR-TB in order to establish a local perspective. Multi-drug resistance is a great terror, and there is a paucity of data from our region related to its associated aspects. Also, we differ genetically when compared to people in rest of the world [9].

Association of good or poor glycemic control with the fate of MDR-TB treatment, if there is any, needs to be demonstrated and elucidated in order to provide an aid to unburden the globe of this devastating ailment.

## Materials and methods

A cohort was conducted prospectively at Chest Medicine (Ward 12), Jinnah Postgraduate Medical Center, Karachi, Pakistan, over a period of six months. Newly diagnosed MDR-TB cases, adults of either gender, were enrolled in the study and were started on a common standardized WHO-recommended treatment regime [2,10].

Permission from the Institutional Ethical Review Committee was taken prior to conducting the study. Informed consent was obtained from all the patients. Demographic information regarding age, gender, literacy, socioeconomic class, and employment status, as well as history of current and past symptoms and duration of TB were taken thoroughly at the time of enrollment. HbA1c (hemoglobin A1c or glycated hemoglobin) was tested at the start of treatment irrespective of previous diabetic status. Three strata on the basis of sugar control were made: normal (HbA1c < 5.6%), impaired (HbA1c: 5.7% to 6.4%), and diabetic (HbA1c ≥ 6.5%). Sputum samples, 30 days apart, were taken from the patients during six months of the initial phase of MDR TB treatment, and patients were labeled as having achieved sputum culture conversion only if two consecutive samples were negative.

Data were analyzed using SPSS Version 20.0 (IBM Corp., Armonk, NY, USA). Mean and standard deviations were calculated for the quantitative variables such as age, height, weight, and HbA1c. Frequencies and percentages were calculated for the qualitative variables. Effect modifiers were controlled through the stratification of age, gender, BMI, smoking status, employment standing, socioeconomic condition, and literacy to see the effect of these on outcome variables. Post-stratification chi-square test was applied taking p-value of ≤0.05 as statistically significant for the analysis of categorical data. Pearson's correlation coefficient was calculated to see the relationship between time to sputum culture conversion and HbA1c.

## Results

Out of the 47 MDR-TB patients, there were 23 (48.9%) males and 24 (51.1%) females between 15 and 65 years of age (mean: 32.81 years; SD: ±3.723). None of the patients were overweight or obese and had BMIs ranging from 12.20 to 21.70 (mean: 16.76; SD: ±2.49). Most (80%) of the patients were non-smokers. Regarding HbA1c, 17 (36.2%) patients fell in the normal category, 17 (36.2%) in the pre-diabetic category, and 13 (27.7%) in the diabetic category (Table 1).

**Table 1:** Demographics of the Patients and the Studied Factors HbA1c, hemoglobin A1c

Variables		Percentage
Gender	Male	48.9
	Female	51.1
Age groups (years)	15-30	50.9
	31-45	27.3
	>45	21.8
Education	Illiterate	45.5
	Primary	27.3
	Secondary	12.7
	Higher	12.7
Socioeconomics (monthly income in PKR)	Lower class group (≤15,000)	54.5
	Lower-middle class (16,000-25,000)	23.6
	Middle class (>26,000-45,000)	20.0
	Upper-middle (46,00-65,000)	1.8
Employment	Non-working dependent	27.3
	Skilled worker/laborer	50.9
	Self-employed	7.3
	Unemployed	14.5
Smoking status	Smoker	20.0
	Non-smoker	80.0
Body mass index (kg/m^2^)	Underweight (<18.5)	52.7
	Normal weight (18.5-24.9)	47.3
Glycemic control (HbA1c in %)	Normal (< 5.6%)	36.2
	Impaired (5.7-6.4%)	36.2
	Diabetes mellitus (HbA1c ≥ 6.5%)	27.7

**Figure 1:**
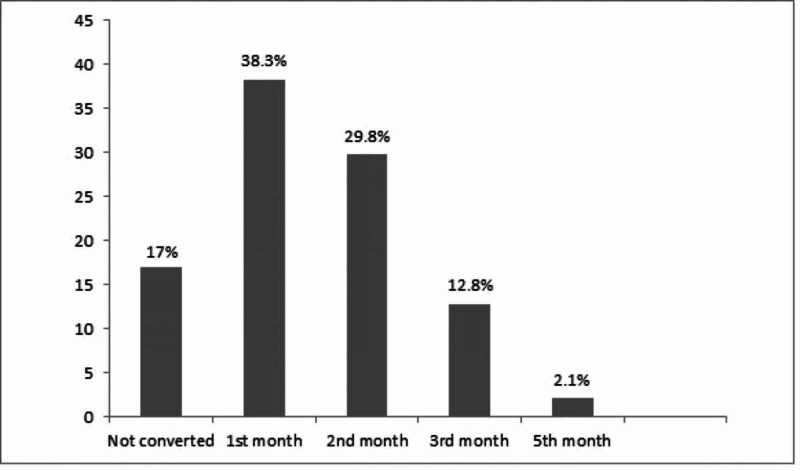
Patients’ Percentage Distribution and Time to Culture Conversion

There were 19 (40.4%) new MDR-TB cases, whereas 28 (59.8%) had taken treatment for DS-TB before. Out of previously treated (for DS-TB) cases, 12 (42.9%) had completed treatment, 12 (42.9%) had failed treatment, and 4 (14.3%) were lost to follow-up. According to drug sensitivity testing results, apart from multi-drug resistance, resistance to fluoroquinolones (pre-XDR) was observed in eight cases and to other drugs in three cases. A total of 39 patients converted during six months, of which 18 (46%) converted in one month, 14 (35.9%) in two months, 6 (15.4%) in three months, and only 1 in five months' time (Figure 1).

**Figure 2:**
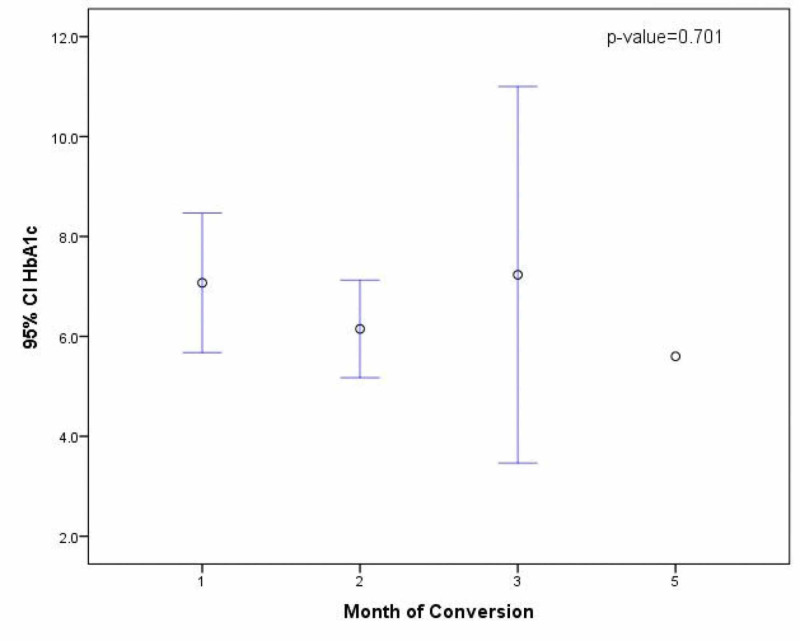
Correlation Between Duration of Sputum Culture Conversion and Glycosylated Hemoglobin Levels

Mean time to sputum culture conversion was 1.77 ± 0.9 months. Those who converted the earliest, i.e., in the first month, were mostly pre-diabetic (44%), and the only patient getting converted in the fifth month had a normal glycemic control (Figure 2).

**Figure 3:**
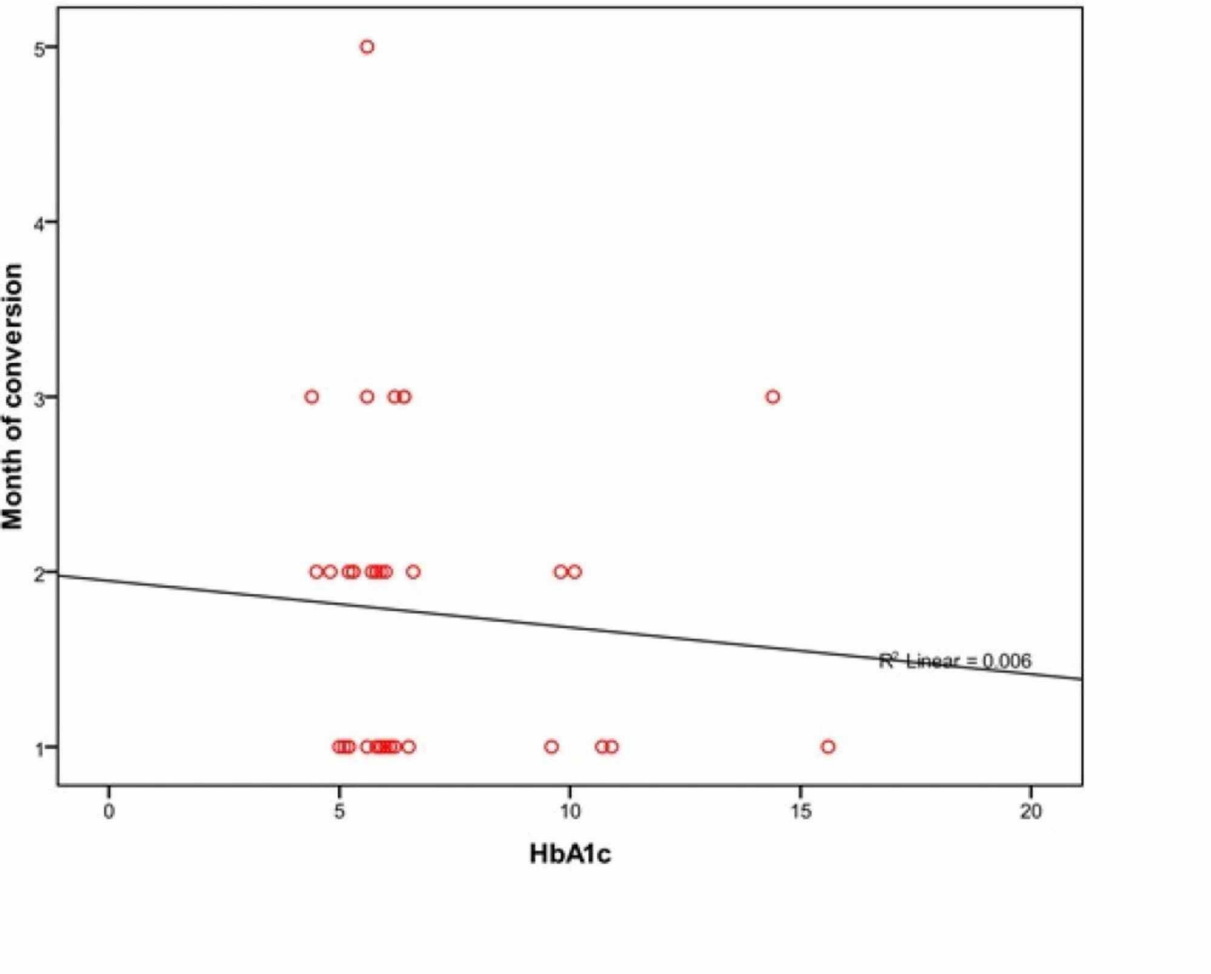
Relationship of Sputum Culture Conversion with Glycosylated Hemoglobin

There was slightly negative correlation between HbA1c and time to sputum culture conversion (r = -0.075, p = 0.649) (Figure 3).

The eight patients who failed to convert during the six months’ period had the following outcomes: three were transferred to another set-up or were lost to follow-up, three died, and two were labeled treatment failures.

## Discussion

In the literature, there have been a number of studies where factors associated with delayed sputum conversion in MDR-TB have been reported. Worse radiological findings especially cavities, resistance to any fluoroquinolone or thioamide, and staying unconverted by the third month were identified as predictors of failure in a study conducted at DOTS-plus projects [11].

Uncontrolled DM or impaired sugar control not only makes an individual susceptible to acquiring infections but also increases the likelihood of suffering from drug resistance in TB [12,13]. Magee et al. looked at the influence of other elements in MDR-TB and concluded that smoking, low body mass index, second-line resistance, presence of lung cavities, and dissemination are contributory but at the same time denied any impact of DM [14]. In an Iranian study, although HbA1c showed an upsurge during TB treatment, the glycemic control did not influence the outcome in DS-TB [15]. The findings of our study in DR-TB subtype were comparable. Therefore, this endorses the crucial contribution of anti-tuberculous drugs than a person’s own immune system in getting rid of both DS and DR types of the disease. The latter, however, might primarily emerge due to poor sugars.

In other researches, median time to sputum culture conversion has shown marked variability from 60 days [16-18] (mostly) to 196 days [19]. It is also observed that the later they convert the more the chances that they experience a poorer outcome as a result of prolonged damage [20]. Our patients showed quite promising results as most of them converted in two months’ time. This highlights the significance of ambulatory care model of treatment suggested by the WHO. The value of careful clinical assessment, identification of treatment supporters, and monthly follow-up assessments, including clinical monitoring, drug compliance, and sputum cultures for monitoring of treatment response cannot be over-emphasized.

The limitation of our study was the inability to track down lost patients and those who were transferred to other set-ups and the strength being one of its kind.

## Conclusions

We conclude that a high proportion, i.e., two-thirds of the MDR-TB population was either diabetic or had impaired sugar control likely showing a connection of glycemic control with drug resistance. There is a slightly inverse correlation between HbA1c and time to sputum culture conversion. A high proportion of MDR-TB patients converted their sputum cultures in up to five months’ period, but most of them were converted in one to two months. This fact clearly stresses the importance of appropriate WHO-recommended regime formation, ensuring drug supplies as well as compliance. More researches of this sort from diversified communities might shed more light on this.
